# Effect of Resonance Breathing on Heart Rate Variability and Cognitive Functions in Young Adults: A Randomised Controlled Study

**DOI:** 10.7759/cureus.22187

**Published:** 2022-02-13

**Authors:** Shyam Chaitanya, Anjum Datta, Bharti Bhandari, Vivek Kumar Sharma

**Affiliations:** 1 Physiology, Government Institute of Medical Sciences, Greater Noida, IND; 2 Physiology, All India Institute of Medical Sciences, Rajkot, Rajkot, IND

**Keywords:** perceived stress, cardiovascular morbidity, cognition, resonance frequency breathing, heart rate variability

## Abstract

Introduction: Heart rate variability (HRV) is an important physiological biomarker of wellbeing, mood, and adaptation, and hence improvements in HRV signify improved health, mood, and adaptation to stress. Resonance breathing has consistently been shown to increase HRV, mood, and adaptability. The study investigated the effects of four-week training of resonance breathing in young adults on HRV, stress, and cognition functions.

Methods: The randomized controlled study was conducted on young men (18-30 years) after obtaining Institutional Ethics Committee approval and written informed consent from the participants. Participants were randomly divided into two groups: the control group and the resonance breathing (RB) group. Baseline parameters, along with heart rate variability, trail tests for cognition, and perceived stress level, were assessed in both groups. After undergoing four weeks of training, the intervention group practiced deep breathing at the resonance frequency for 20 minutes per day for four weeks, whereas the control group did not. All parameters were recorded again after four weeks of resonance breathing.

Results: No difference was observed in the HRV parameters in the control group at baseline and four weeks after the baseline recording (p>0.05). In the intervention group, there was a significant improvement in the HRV parameters, the standard deviation of the normal-to-normal interval (SDNN), the proportion derived by dividing NN50 by the total number of NN intervals (pNN50), and total power, after practicing four weeks of resonance breathing (p<0.05). A significant difference in these parameters was also observed in the control and intervention groups after four weeks (p<0.05). There was a significant improvement in the Trails A and B test performance in the intervention group after practicing for four weeks of RB. Similarly, the perceived stress score decreased significantly in the RB group in comparison to its baseline value as well as the control group value.

Conclusion: Increased parasympathetic and decreased sympathetic activity were observed after practicing 20 minutes of resonance frequency breathing every day for four weeks. It also improved cognition and reduced perceived stress levels among young adults. It is recommended that young adults should practice deep breathing at the resonance frequency for a few minutes every day. This would allay anxiety and stress, improve their cognitive performance, and also reduce their cardiovascular morbidity.

## Introduction

Urbanisation, changing lifestyles, and competitive streaks in all spheres of life have made anxiety, stress, depression, and resultant psychosomatic illnesses an inevitable part of human life. Stress and autonomic dysfunction are the common risk factors for future cardiovascular disease (CVD) and also negatively impact cognitive functions, which are often overlooked [1]. HRV is an index of the autonomic balance of an individual defined as oscillations between consecutive heartbeats, and it is considered a physiological phenomenon [2]. Heart rate variability (HRV) is a non-invasive physiologic measure of autonomic function that facilitates the identification of people at the risk of developing cardiovascular complications [3]. Dysfunctional regulation of the hypothalamic-pituitary-adrenal (HPA) axis has been identified as an important biological mechanism underlying stress-related diseases [4]. Heart rate increases during inhalation and decreases during exhalation in a respiratory cycle and this phenomenon is called respiratory sinus arrhythmia (RSA) [5]. Heart rate variability biofeedback (HRVBFB) or resonance breathing is breathing at a slow rate, usually 4.5 to 7 breaths per minute, which depends on each individual, to maximise their RSA [6]. Self-training in resonance breathing lowers stress, blood pressure, and improves mood [7]. Training on resonance breathing improves vagal tone, thereby improving HRV, which is an index of stress and health [8]. Enhanced vagal tone improves cognitive abilities based on the neuro-visceral integration model [9]. Self-training in resonance breathing, mindfulness meditation, and aerobic exercise for five weeks has been shown to improve cognitive functions such as attention control and executive function in young adults [10]. Resonance breathing reduces depression and anxiety through its vagal pathway, affecting the locus coeruleus, orbitofrontal cortex, amygdala, insula, and hippocampus [11]. Studies on the effect of four weeks of training of resonance breathing training in young adults on HRV and cognition functions are sparse. Hence, we have planned the present study.

## Materials and methods

The study commenced after obtaining approval from the Government Institute of Medical Sciences - Institutional Ethics Committee (GIMS-IEC). Written informed consent was obtained from all the participants.

This is a randomised control trial. Students of neighbouring universities and relatives of patients who attended OPD were included in the study. The sampling was done on the principle of a randomised controlled trial. A convenient sample of 50 subjects was taken from the specified age group (18-30 years). The subjects were randomised into two groups of 25 each, using a computer-generated random number as follows. (i) Experimental group: subjects were given supervised training of 20 minutes for breathing at the resonance frequency for eight sessions, i.e., two in each week, and were asked to practice at home in the morning. After four weeks of training, they practiced 20 minutes of resonance breathing for a period of another four weeks. (ii) Controlled group: subjects were allowed to take normal breathing hence no intervention was given.

Inclusion criteria were (i) young, apparently healthy males in the age group of 18 to 30 years, possessing smart phones and (ii) consenting to participate. Exclusion criteria were (i) individuals involved in any regular exercise, sports, yoga, or meditation and are on any special weight reducing diet plan; (ii) individuals who have any physical disability to perform breathing exercise, visual problems including colour defects, any major physical systemic or psychiatric illness and those on any regular medication; (iii) individuals, who smoke, consume alcohol, use any stimulants and recreational drugs; (iv) individuals with BMI less than 18 kg/m^2^ or more than 25 kg/m^2^.

Method of recruitment and allocation

Subjects were recruited from one of the neighbouring universities and relatives of patients admitted to the hospital. The experimental group (n=25) was asked to practice resonance breathing for 20 minutes once a day for a period of four weeks after supervised and unsupervised training sessions spanning over four weeks.

Resonance breathing protocol

In the autonomic testing (AFT) lab, the resonance-breathing rate was determined using the protocol given by Lehrer and Gevirtz [2]. Each recruited subject was instructed to visit the AFT lab, Department of Physiology, GIMS, between 7 and 9 AM for a maximum of one hour after a restful sleep and without breakfast or tea/coffee for baseline recording and determining the resonance breathing rate. They were instructed to abstain from any strenuous exercise 24 hours prior.

After familiarisation training with the equipment and the procedure for HRV, a brief history was obtained, and a physical examination was carried out. This was followed by the determination of their own resonance-breathing pattern through HRV recording.

While recording the HRV using a standard three-lead ECG placed on their torso, the subjects were asked to breathe for two minutes at a given frequency, starting from 4.5 to 7 breaths per minute with an increment of 0.5 breaths per minute, pacing with the Android application "Paced Breathing," trying to keep the depth of the breathing approximately constant. The breathing rate at which their HRV (total power) was maximum was considered the resonance-breathing rate for that individual. After obtaining their resonance-breathing rate, the Android App "Paced Breathing" was installed on their mobiles and they were trained for resonance breathing. During the training sessions, the participants in the control group practiced resonance breathing for 20 minutes in the evening in the physiology department, supervised by the investigator; only eight supervised sessions were conducted during the training period. They were asked to practice RB (without supervision) in the morning. Throughout the training, they were cautioned to take shallow and natural breaths and avoid hyperventilation. Once trained, they were instructed to practice resonance breathing every day in the morning before breakfast or coffee for 20 minutes and enter it in a logbook. This was done for a period of four weeks. Sending reminders to each participant and contacting them when required ensured compliance.

Recorded parameters

Baseline Physiological Parameters

Age, arterial blood pressure, heart rate, height, and weight were recorded. Body mass index (BMI) was calculated by weight (kg) divided by the square of height (m) (Quetelet’s Index).

Heart Rate Variability

The subject was asked to lie comfortably on a couch and relax for 10 minutes. ECG recording was done for 10 minutes for short-term HRV analysis by following the standard procedure as recommended by the task force on HRV [12]. HRV was assessed using time-domain and frequency-domain analysis. An electrocardiogram was acquired using a computer-based digital data acquisition system using PowerLab® electrocardiographs (ADIntruments, Sydney, Australia). Recordings were captured and stored by LabChart® v. 8.0 software (ADIntruments), with a sampling rate of 500 Hz and 1 ms time resolution. All artifacts were reviewed by visual inspection on the computer display. Only segments with >90% pure sinus beats were included in the final analysis. The final analysis of HRV was done using the in-built Kubios HRV® software (version 2.2, Helsinki, Finland). Sampling rates for acquiring signals were set at 1 kHz in order to have sufficient time precision in detecting the changes in all the parameters.

HRV was recorded at baseline and eight weeks later in both groups (four weeks of training and four weeks of resonance breathing practice in the experimental group).

Cognitive Function

Paper and pencil cognitive tests were used to assess cognitive function; these were the Trail Making Tests parts A (TMT-A) and B (TMT-B) [13]. The TMT-A was used to assess visual-motor speed and attention. The participants were instructed to draw a straight line to connect 25 consecutive numbered circles. The score was the time taken (in seconds) to complete the task. In TMT-B, the participants were instructed to draw lines connecting numbers and letters in order, alternating numbers and letters. A practice trial was administered to the participants before the start of the test. The score was the total time (in seconds) taken to complete the task. The time for the tests was measured with a stopwatch. The result was interpreted as given in Table 1. In both groups, the tests were performed twice, at baseline and after eight weeks.

**Table 1:** Interpretation of trail making tests

	Average	Deficient	Rule of thumb
Trail A	29 s	>78 s	Most in 90 second
Trail B	75 s	>273 s	Most in 3 minute

Perceived Stress

The perceived stress was measured using a pre-validated freely available scale, the Perceived Stress Scale (PSS) scale given by Cohen et al. [14]. The scores were interpreted as shown in Table 2.

**Table 2:** Interpretation of Perceived Stress Scale score

Score range	Stress level
0-13	Low stress
14-26	Moderate stress
27-40	High perceived stress

**Figure 1:**
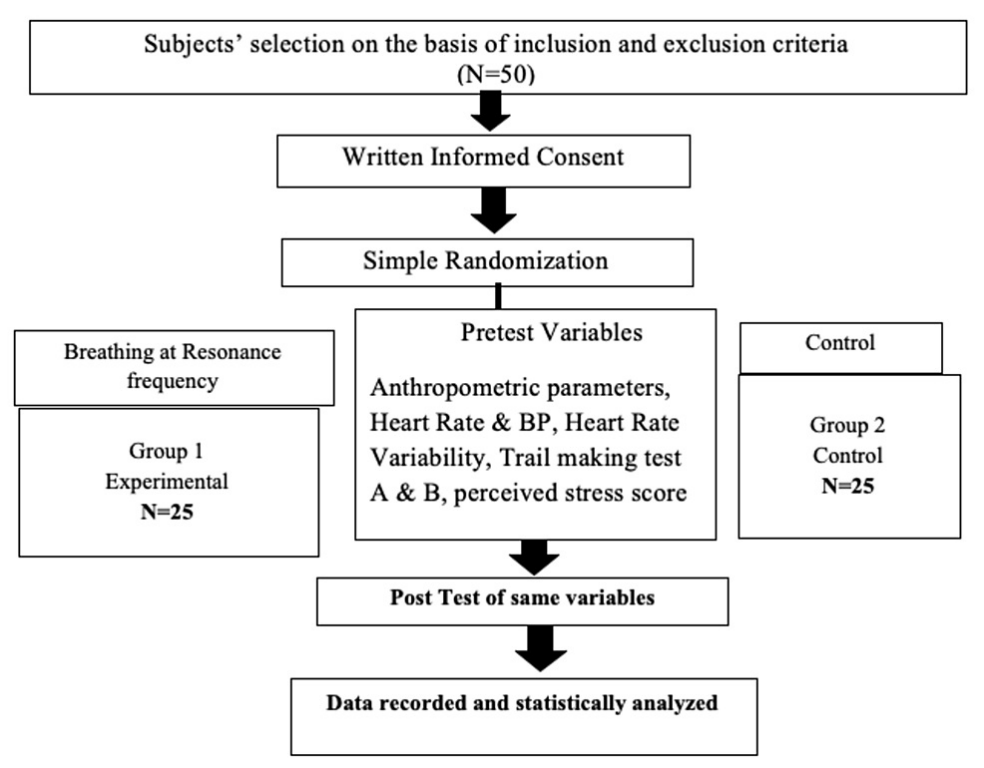
CONSORT flow chart of the methodology

The perceived stress was measured twice, at baseline and after eight weeks, in both the groups (Figure 1).

Data management

Data were analysed using SPSS software (IBM Corp. Released 2017. IBM SPSS Statistics for Macintosh, Version 25.0. Armonk, NY: IBM Corp). Data were tested for normality and expressed as mean ± standard deviation. For intergroup comparison, unpaired student’s t-test or Mann-Whitney-U test was used. For intra-group comparison, paired t-test or Wilcoxon Signed Rank test was used depending on data distribution. The significance level of p<0.05 was used in the study.

## Results

Demographic and baseline characteristics

No significant differences were observed in any of the demographic and baseline parameters of the participants (p>0.05). The demographic and baseline characteristics are shown in Table 3.

**Table 3:** Demographic and baseline characteristics of the participants Data are presented as mean ± SD. BMI: body mass index; SBP: systolic blood pressure; DBP: diastolic blood pressure; MBP: mean blood pressure; HR: heart rate. P<0.05 was considered statistically significant. Intergroup comparison was done using an unpaired t-test.

S. No.	Parameters	Control group (n=25)	RB group (n=25)	p-value
1.	Age (years)	23.5±3.43	24±3.11	0.45
2.	Height (cm)	166±6.78	168.86±6.12	0.39
3.	Weight (kg)	69.78±9.19	74.76±9.99	0.55
4.	BMI (kg/m^2^)	23.37±3.03	25.78±3.99	0.7277
5.	SBP (mmHg)	128.39±19.06	124.45±17.89	0.99
6.	DBP (mmHg)	74.78±6.78	73.32±6.12	0.85
7.	MBP (mmHg)	88.34±15.06	92.37±11.21	0.76
8.	HR (BPM)	83.76±3.39	74.56±2.56	0.98

Heart rate variability

At baseline and four weeks after the baseline recording (p>0.05), no difference was observed in the HRV parameters in the control group. In the intervention group, there was a significant improvement in the HRV parameters (SDNN, pNN50, and total power) after practicing four weeks of resonance breathing regularly (p<0.05). A significant difference in these parameters was also observed in the control and intervention groups after four weeks (p<0.05). The values of HRV parameters in the two groups before and after intervention are given in Table 4.

**Table 4:** Heart rate variability parameters in the two groups Data are presented as mean ± SD. SDNN: standard deviation of the normal-to-normal interval, RMSSD: the square root of the mean of squares of the differences between adjacent NN intervals, pNN50: the proportion derived by dividing NN50 by the total number of NN intervals, LF: low-frequency power (ms^2^), HF: high-frequency power (ms^2^). P<0.05* was considered statistically significant. The intragroup comparison was done using the Wilcoxon Signed-Rank test and intergroup comparison was done using the Mann-Whitney U test.

S.No.	Parameters	Control group (n=25)		p-value	RB group (n=25)		p-value	Intergroup p-value
		Pre	Post		Pre	Post		
1.	SDNN (ms)	63.44±47.24	61.42±18.74	0.40	66.69±33.03	78.76±24.15	0.001*	0.001*
2.	RMSSD (ms)	74.97±57.09	72.30±28.06	0.45	56.66±27.46	74.45±22.99	0.96	0.66
3.	pNN50 (ms)	31.49±31.49	48.66±18.79	0.99	30.39±30.39	54.82±14.49	0.02*	0.05*
4.	LF (ms^2^)	1141.10±1509	1127.76±1315	0.34	1587.6±2308	2546.16±2119	0.04*	0.003*
5.	HF (ms^2^)	1895.37±1765	1543.246±1367	0.44	2715.1±2466	3067.62±2502	0.51	0.06
6.	LF/HF	0.61±0.4311	0.65±0.2370	0.856	2.05±1.889	0.25±0.1323	0.02*	0.006*
7.	Total power	3751.52±2347	4009.6±2056	0.73	3570.23±2829	5543.38±2838	0.002*	0.05*

Cognitive function and perceived stress level

There was a significant improvement in the Trail A and Trail B test performance in the intervention group after practicing four weeks of resonance breathing. However, no difference was there at baseline. Similarly, the perceived stress score decreased significantly in the RB group in comparison to its baseline value as well as the control group value. These parameters are given in Table 5.

**Table 5:** Trail test score and perceived stress among the participants Data are presented as Mean ± SD. P<0.05* was considered statistically significant. Intragroup comparison was done using Wilcoxon Signed-Rank test and intergroup comparison was done using Mann-Whitney U test.

S.No.	Parameters	Control group (n=25)		p-value	RB group (n=25)		p-value	Intergroup p-value
		Pre	Post		Pre	Post		
1.	Trail A (s)	27.78±4.199	27.52±4.870	0.46	27.82±	23.34±	0.03*	0.04*
2.	Trail B (s)	64.34±9.321	63.08±9.123	0.726	63.39±	53.52±53.12	0.01*	0.02*
3.	Perceived stress score	26.56±21.34	25.86±24.31	0.44	27.65±	21±20.09	0.01*	0.04*

## Discussion

The study assessed the effects of breathing at resonance frequency on autonomic activity, cognitive activity, and perceived stress. It was hypothesised that four weeks of resonance breathing for 20 minutes each day would improve sympathovagal balance, cognitive functions, and reduce perceived stress in healthy adults. Improvement in the parasympathetic activity was observed on practicing RB in the intervention group, as shown by increased SDNN and pNN50. There was a significant improvement in the scores in the intervention group on Trail tests, as well as a significant decrease in the perceived stress score in the RB group when compared to the pre-RB values and control group values.

The current study was conducted on male subjects to avoid the effect of gender on heart rate variability. Gender differences in HRV have been observed in normal as well as diseased conditions [15,16]. Women in the specified age group have a higher resting parasympathetic tone in comparison to men [15].

In the current study, the RB rate was fixed for each participant by following a standard protocol [2]. In all the participants, it was found to be 6-6.5 breaths per minute. At this rate, the changes in the time domain and frequency domain parameters of HRV were suggestive of parasympathetic predominance. It has been postulated that breathing at a rate of close to six breaths/minute yields the highest amplitude of oscillations in HRV, and respiration and heart rate oscillations are in phase with each other [2]. Resonance frequency breathing is an integral component of HRV biofeedback [7-9]. It has been demonstrated that breathing at 6 breaths/minute as part of yoga exercise improves oxygen saturation and baroreflex sensitivity [17]. Some researchers have also studied the positive effects of resonance breathing at lower rates on HRV. Lin et al. suggested that breathing at a rate of 5.5 breaths per minute with an equal inhalation-to-exhalation ratio increases heart rate variability [18]. However, in asthma patients, it was observed that heart rate and breathing are not always in phase during resonance frequency breathing [19].

Improvement in the Trail tests after practicing RB for a duration of four weeks was observed in the RB group. The literature review could not retrieve any articles that have studied the effect of RB on cognition, although many researchers have established a direct association between improved HRV and cognition [20,21]. In one study, it was found that participants with higher HRV showed better performance on cognitive tasks than those with low HRV [20]. A systematic review by Forte et al. has indicated that HRV can be used as an early biomarker of cognitive impairment in a healthy population [21]. It is postulated that better cognitive performance in the intervention group in the study can be recounted to improved HRV following four weeks of resonance breathing training. In the current study, the perceived stress level was found to decrease significantly after four weeks of RF breathing training. Similar to this study, Steffan et al. have proved that RF breathing elevates current mood [7]. In a recently published meta-analysis of 24 studies, it was established that breathing at resonance frequency reduced self-reported stress and anxiety with a large effect size [22]. It was further suggested that it had benefits even for those without clinical levels of anxiety. It has been postulated that increasing vagal activity increases physical and emotional resilience. Hence, improving vagal activity by RF breathing intervention for four weeks may be the possible cause of the improved perceived stress score in the intervention group participants. Further, Mather and Thayer proposed that high-amplitude oscillations in heart rate produced by resonance breathing modulate brain oscillatory activity in brain regions associated with emotion regulation [23]. 

The improvement in cognition and perceived stress by resonance breathing can further be explained by the Neuro-Visceral Integration Model, rationalised by Thayer et al. [24]. This substantiates the functional relationship between the prefrontal cortex and the heart via the central autonomic network (CAN). The CAN includes structures like the anterior cingulate gyrus, ventromedial pre-frontal cortices, the central nucleus of the amygdala, the periaqueductal grey matter, the nucleus tractus solitarus, the nucleus ambiguus, the ventro-lateral/medial medulla, etc., involved in stress-regulation, emotional and cognitive responses. All these structures form connections among themselves, and their output impacts the neuro-vegetative pathways between the heart and brain.

This study included only young men (18-30 years); hence, it is difficult to say if findings can be extrapolated to other age groups and women. We could not assess baroreflex sensitivity. Future research could focus on the assessment of baroreflex sensitivity to understand the pathways leading to HRV changes. One of the strengths of the study was its randomised controlled design, thus minimising the chances of the alterations observed after intervention being due to random effects.

## Conclusions

In the current randomised controlled study, we conclude that 20 minutes of resonance frequency breathing every day for four weeks may lead to positive changes in HRV, i.e., increased parasympathetic and decreased sympathetic activity. It may also improve cognition and reduce the perceived stress level among young adults. It is recommended that young men should practice deep breathing at the resonance frequency for a few minutes every day. This would allay anxiety and stress, improve their cognitive performance, and also reduce their cardiovascular morbidity.
